# OA−ICOS−Based Oxygen and Carbon Dioxide Sensors for Field Applications in Gas Reflux Chicken Coops

**DOI:** 10.3390/s25030886

**Published:** 2025-01-31

**Authors:** Weijia Li, Guanyu Lin, Jianing Wang, Jifeng Li, Yulai Sun, Depu Yao, Xiaogang Yan, Zhibin Ban

**Affiliations:** 1 Changchun Institute of Optics, Fine Mechanics and Physics, Chinese Acafemy of Sciences, Changchun 130033, China; lwj1178607860@163.com (W.L.); linguanyu1976@163.com (G.L.); lijifeng@ciomp.ac.cn (J.L.); breadovski613@163.com (Y.S.); dpyao_whu@163.com (D.Y.); 2 University of Chinese Academy of Sciences, Beijing 100049, China; 3 Northeast Agricultural Research Center of China, Jilin Academy of Agricultural Sciences, Gongzhuling 136199, China; yanxiaogang1977@163.com (X.Y.); banzb0620@163.com (Z.B.)

**Keywords:** OA−ICOS, gas sensor, response time, respiratory entropy

## Abstract

To facilitate the effective assessment of respiratory entropy during poultry breeding, a novel oxygen (O_2_) and carbon dioxide (CO_2_) sensor was developed based on the off−axis integrated cavity output spectroscopy technique, featuring effective absorption optical paths of 15.5 m and 8.5 m, respectively. The sensor employs integrated environmental control technology, substantially enhancing detection precision. To improve the instrument’s response speed, the miniaturization of the cavity and structural optimization were implemented, achieving a rapid response time of merely 6.22 s, addressing the stringent requirements for quick responsiveness in poultry respiration thermometry research. A signal processing model tailored for on−site applications was designed, boosting the system’s signal−to−noise ratio by 4.7 times under complex environmental noise conditions. Utilizing Allan variance analysis, the sensor’s detection limits for O_2_ and CO_2_ were ascertained to be 2.9 ppm and 7.4 ppb, respectively. A 24−h field application test conducted in Gongzhuling demonstrated that the sensor’s results align with the respiratory characteristics of poultry under normal physiological conditions, validating its extensive potential for application in respiratory analysis, environmental monitoring, and industrial sectors.

## 1. Introduction

Poultry, as a crucial food source for humans, has consistently been the focus of research aimed at enhancing both yield and quality. However, the assessment of the absorption and conversion processes of essential nutritional elements such as carbon and nitrogen in poultry is challenging due to the limitations of current observation methods in avian husbandry. Presently, the measurement of gas production and consumption during feeding, combined with the calculation of the respiratory quotient through indirect calorimetry, provides insights into the nutritional intake and digestive status of poultry, playing a vital role in observing the process of nutrient conversion and promoting increased and improved production [1,2,3].

Among the various gas detection methods, electrochemical [4], gas chromatography [5], and optical techniques are commonly employed [6]. In Table 1, the performance parameters and prices of the four gas measurement methods are compared. The electrochemical method is predominantly used in poultry husbandry observations; however, issues such as zero−drift, limited precision, and gas poisoning have constrained its application [7]. In contrast, the G2207i O_2_ analyzer developed by Picarro, utilizing Cavity Ring−Down Spectroscopy (CRDS) [8], offers significantly enhanced precision in concentration detection, but its high cost renders it impractical for agricultural research. Indirect measurement methods are limited by a short measurement duration of only 20 min in the metabolic process of poultry. In practical applications, not only is precision crucial, but also a rapid response time. Consequently, OA−ICOS technology has emerged as a more advantageous detection technique, given its ability to meet the criteria of precision, response time, and cost−effectiveness.

Compared to CO_2_ measurement, research on O_2_ measurement is relatively scarce as it faces the challenges of wide range and high precision detection. The sensors commonly used in the field still primarily rely on electrochemical methods. In 1989, Dr. Bradley Joseph and colleagues designed an O_2_ sensor array based on semiconductor manufacturing using the electrochemical approach [9]. While these sensors are cost−effective and compact, the widespread issues of significant offset and long response times associated with electrochemical sensors make them inadequate for certain demands. In optical measurement methods, recent research, including that of Qixin He [10], has achieved a measurement precision of 199 ppm and a response time of 30 s using the OA−ICOS gas concentration detection method and pressure calibration with the PSO−SVM algorithm. Nevertheless, this falls short of the precision and response time required for agricultural detection. In light of the current research, the design and implementation of a low−cost, high−precision, and rapid−response O_2_ and CO_2_ sensor is an urgent issue that needs resolution.

This study addresses the core requirements of high precision, rapid response, and cost−effectiveness in gas sensors for the poultry industry. It introduces a dual−gas sensor based on OA−ICOS technology, which significantly improves the measurement accuracy of O_2_ and CO_2_ concentrations, achieving 2.9 ppm and 7.4 ppb (Allan variance), respectively, through structural, adjustment, and environmental control and filter comparisons. Moreover, a compact integral cavity with a length of 6 cm and a diameter of 2 cm was designed to provide a rapid response time of 6.22 s. The sensor utilizes DFB butterfly lasers at 760.9 nm and 2004 nm, simulates the absorption cross−sections of the two gases using the HITRAN database, and identifies the absorption spectral lines for O_2_ and CO_2_ at 760.654 nm and 2004 nm, respectively. An off−axis integral cavity with effective optical paths of 15.5 m and 8.5 m is designed. The sensor employs wavelength modulation spectroscopy (WMS−2f) for signal amplification. The calibration of the sensor is performed using a gas mixer, and it is applied within a novel multi−chamber parallel open−loop poultry respiratory calorimetry device to measure the O_2_ consumption and CO_2_ production of different chicken breeds. These data are then used to assess physiological activity and feeding information through the respiratory quotient calculated using indirect calorimetry [11].

## 2. Materials and Methods

### 2.1. Sensor Solutions

**Figure 1:**
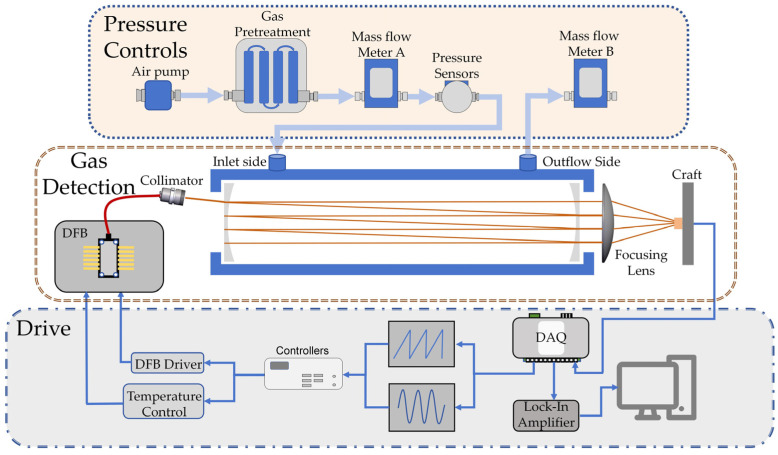
O_2_ detection system principle.

The sensor scheme is exemplified by an O_2_ single−channel measurement system, with Figure 1 illustrating its detection principle. This system employs a 14−pin butterfly−packaged Distributed Feedback (DFB) laser diode as the light source, utilizing Tunable Diode Laser Absorption Spectroscopy (TDLAS) in the vicinity of 760.9 nm for wavelength scanning. An optical collimator (FC/APC−780, Thorlabs, Newton, NJ, USA) is utilized at the input end of the integrating cavity for beam collimation. The high−precision integrated cavity, approximately 6.2 cm in length, consists of two concave high−reflection mirrors with a reflectivity of 99.6%, achieving an effective absorption path length of 15.5 m. To ensure the coaxial alignment of the internal mirrors and to minimize the impact of temperature on the cavity’s stability, indium steel is specifically chosen as the cavity material. At the cavity’s output end, a 100 mm focal length lens focuses the beam onto the photosensitive surface of a photodiode (PDAPC1, Thorlabs), with data acquisition and driving equipment (DAQ, USB−6211, National Instrument, Austin, TX, USA) used for data collection. To mitigate the impact of environmental factors on the device’s performance, a dual mass flowmeter is adopted for pressure and flow stabilization of the measured gas, while the gas cavity structure is maintained at a constant temperature via a thermoelectric cooler (TEC, SiRui, Zhongshan, China) [12,13,14].

### 2.2. Absorption Spectra and Modulation

**Figure 2:**
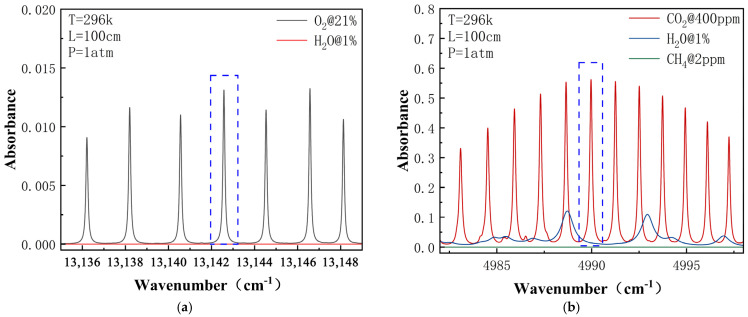
The simulated detectable absorption lines of (**a**) O_2_ and (**b**) CO_2_ at room temperature and the atmospheric pressure in the region with possible interference gases. The blue dotted line enclosed area represents the gas absorption peaks selected by this article.

For practical application considerations, this scheme focuses on spectral line intensity, the absence of interference from other gases, and compatibility with mature laser technology. Based on the HITRAN database [15], a simulation analysis of the absorption spectral lines of O_2_ and CO_2_ under standard temperature and pressure conditions is conducted (Figure 2). Common interfering gases such as water vapor (H_2_O) and methane (CH_4_) present minimal interference in the 13,142.2 cm^−1^ waveband for O_2_ detection, and meet the requirements for extensive O_2_ concentration measurements; interference from water vapor (H_2_O) on CO_2_ is relatively weak, with its absorbance at least three times lower than that of CO_2_, allowing for elimination through subsequent data processing. Therefore, the chosen absorption wavebands are 13,142.2 cm^−1^ (760.89 nm) for O_2_ and 4990 cm^−1^ (2004 nm) for CO_2_.

**Figure 3:**
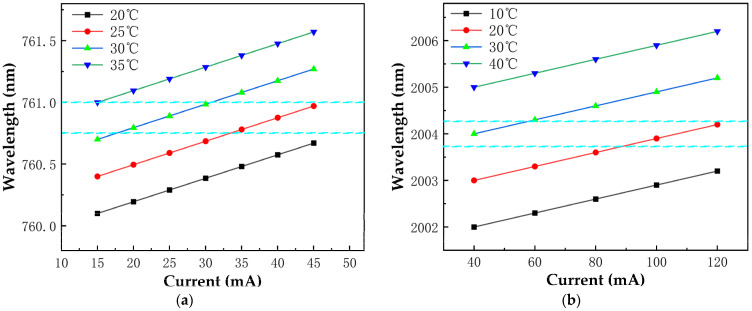
Emission spectrum performance test results of (**a**) O_2_ and (**b**) CO_2_ lasers for optimized driving condition selection. The blue dashed area is the region where the laser modulation is carried out, covering the gas absorption peak range.

Regarding laser wavelength modulation, the center current for the O_2_ laser diode is set at 26.5 mA, with a temperature of 26 °C, a modulation frequency of 2000 Hz, and a modulation depth of 1 mA; for the CO_2_ laser diode, the center current is 61.5 mA, the temperature is 22 °C, and the modulation frequency and depth are 2000 Hz and 5 mA, respectively (Figure 3) [16,17,18].

### 2.3. Light Path Analysis and Integrating Cavity Setting

**Figure 4:**
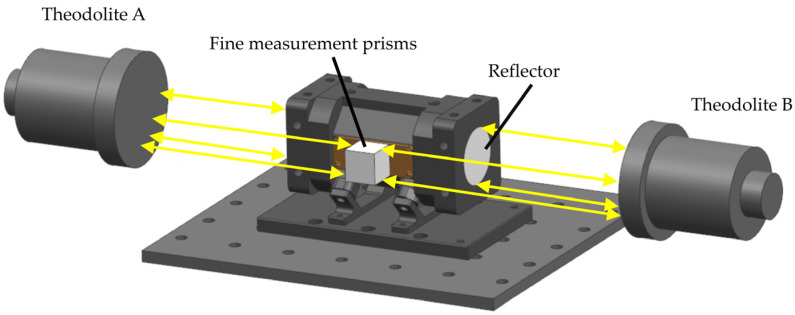
The method of mounting concave mirrors for the coordinate transformation of the latitude and longitude instruments.

In the practical testing of optical path analysis and integrated cavity adjustment, the reflectivity of concave lenses and their assembly precision play a decisive role in the effective optical path of the integral cavity [19]. To ensure measurement accuracy, this scheme strives to conduct measurements within a linear range, thus safeguarding sensor performance. The reflectivity was determined to be 99.6% for O_2_ and 99.27% for CO_2_, with a spherical lens of 1000 mm curvature utilized to construct an integral cavity structure of 6.2 cm. According to the optical path calculation method from the literature [20], the effective absorption optical paths measured were 15.5 m and 8.5 m, respectively. In terms of assembly, the key lies in ensuring that the two concave mirrors are relatively parallel in the same plane along the same axis [21,22]. Compared to traditional visible laser integrator cavity assembly methods, this proposal advocates the use of precision prisms to establish a three−dimensional structural coordinate system and employs a theodolite to adjust the integrator cavity mirror seats, thus quantifying the relative position of the two−dimensional mirrors with an accuracy of up to 30 arc seconds (Figure 4).

### 2.4. Temperature and Pressure Control

**Figure 5:**
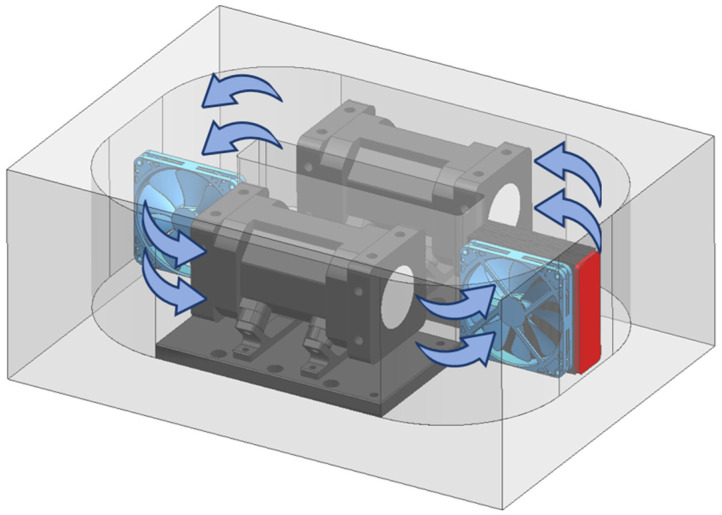
Integral cavity ring temperature control.

**Figure 6:**
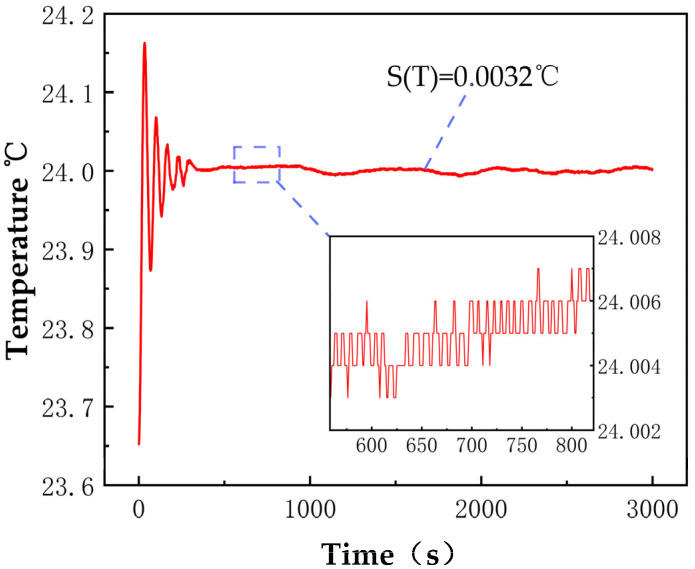
Temperature control measured data.

Temperature and pressure are critical factors affecting detection accuracy. In this study, a specialized temperature and pressure control unit was designed. An independent constant temperature cavity was designed within the temperature control section (Figure 5), with the most thermally sensitive cavity placed inside a constant temperature box as the primary control target. The constant temperature box is made of a thermally less sensitive EEP (expanded polypropylene) material and is doubly wrapped with aluminum alloy metal films to minimize the impact of external temperature fluctuations on performance. The temperature control unit utilizes temperature and pressure sensors within the cavity for closed−loop control feedback. Temperature regulation is achieved by controlling the direction and magnitude of current flow through Thermoelectric Coolers (TECs) for heating or cooling the air inside the constant temperature box. A looped unidirectional heat flow exchange method is adopted to avoid turbulence within the temperature−controlled cavity, and key positions within the cavity employ multi−point temperature measurement combined with weighted temperature control to ensure precision as much as possible. The temperature control accuracy of critical positions within the cavity can reach ±0.005 °C, as demonstrated by the actual measurement results shown in the Figure 6.

**Figure 7:**
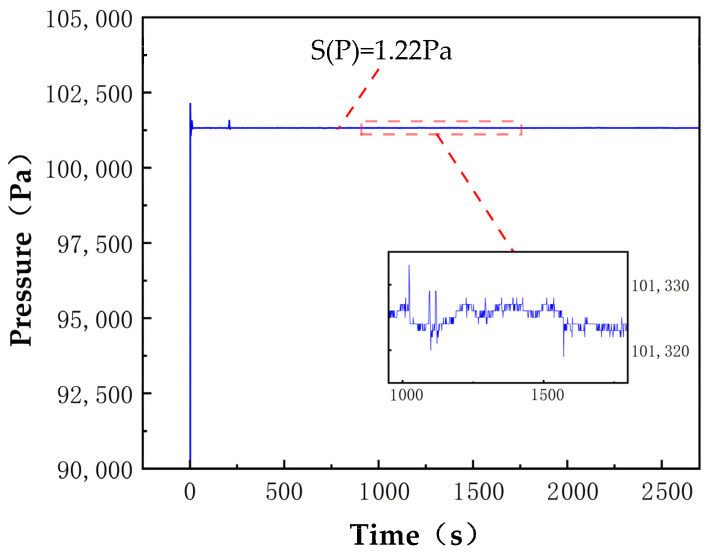
Pressure control measured data.

For pressure control, the pressure sensor is connected to the measurement gas chamber, converting pressure signals into digital signals and transmitting them to the signal processing module. The flow control module feeds back current flow information to the signal processing module and works in conjunction with the PID control module. Based on the comparison between the measured pressure and the set pressure values, it outputs the corresponding flow control information. Through digital−to−analog conversion, a proportional solenoid valve controls the opening flow, achieving precise control of the cavity pressure. The pressure inside the cavity is adjusted by mass flow meters at the inlet and outlet ends of the cavity. The temperature and pressure control unit developed in this study can achieve a stability of ±0.005 °C and ±1.5 pa (Figure 7).

## 3. Results

### 3.1. Gas Preparation

To enhance the accuracy of the sensor measurements, a gas preparation system was configured to produce multiple concentration gas samples with a stable output flow rate. Standard gases of 50% O_2_ and 3% CO_2_ concentrations have an uncertainty of ≤1%. A gas mixer (SX−7100, LNI Swissgas, Versoy, France) was used to thoroughly mix pure nitrogen with standard gases, preparing multi−gradient standard gases. The accuracy of the gas mixer is better than 0.5%, and its repeatability exceeds 0.2%.

### 3.2. Calibration and Data Fitting

Multiple standard concentrations of O_2_ and CO_2_ samples were tested to establish the relationship between the 2f amplitude and gas concentration. Sampling intervals of 5 min were used to obtain an average, and concentration calibration was conducted using linear fitting.

**Figure 8:**
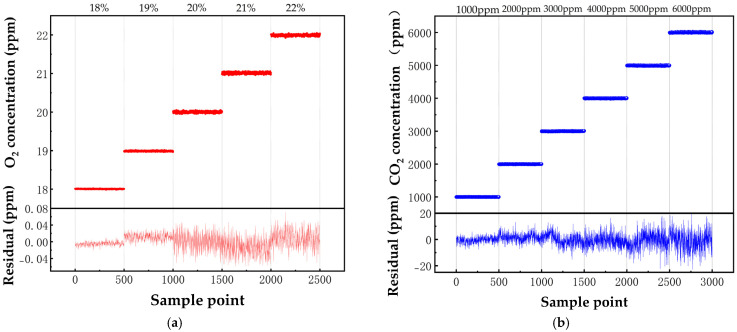
(**a**) O_2_ experimental data at different concentrations with fitted residuals; (**b**) experimental data and fitted residuals for CO_2_ at different concentrations.

As shown in Figure 8, the fitting equation for O_2_ is Y = 6.806 × 10^−7^ × X − 15.59, with a correlation coefficient R^2^ = 0.9973; for CO_2_, the fitting equation is C = 0.002697 × U + 242.7, with a correlation coefficient R^2^ = 0.9998. After fitting, residuals were calculated and the maximum deviation between the two was estimated. The relative concentration deviation of O_2_ was approximately 0.6%, and that of CO_2_ was 0.7%.

### 3.3. Allan Variance Estimation

After ensuring that the temperature and pressure conditions were met, the Allan variance method was employed to evaluate the long−term stability and detection limits [23]. By allowing 99.99% nitrogen to flow continuously into the gas chamber for at least 15 min, the relationship between the Allan variance and integration time was observed.

**Figure 9:**
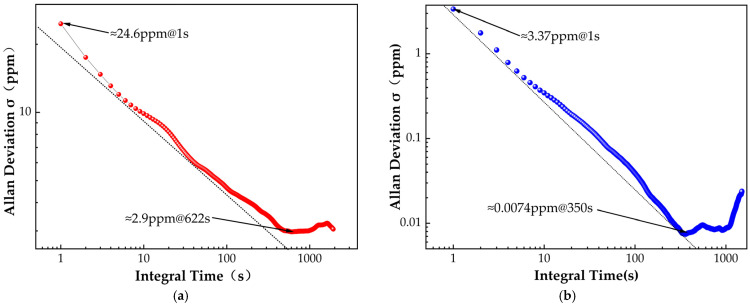
(**a**) O_2_ and (**b**) CO_2_ measurements of a sensor system based on Allan variance estimation in pure N_2_ gas for 20 min.

Figure 9a shows the detection situation for O_2_, where the minimum detection limit reaches 24.6 ppm at an integration time of 1 s, and the optimal detection limit is 2.9 ppm at 622 s. In comparison, the measurement range for CO_2_ is smaller, but with higher precision; the detection limit is approximately 3.37 ppm at 1 s and as low as 7.4 ppb at 350 s.

### 3.4. Response Time

**Figure 10:**
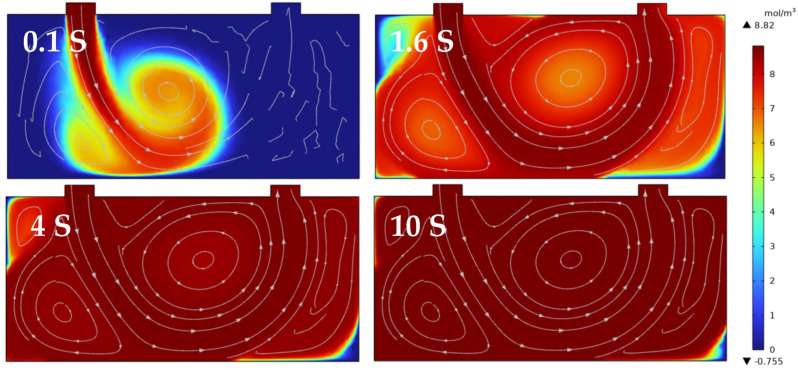
Conventional off−axis integral cavity structure internal fluid simulation and response time.

**Figure 11:**
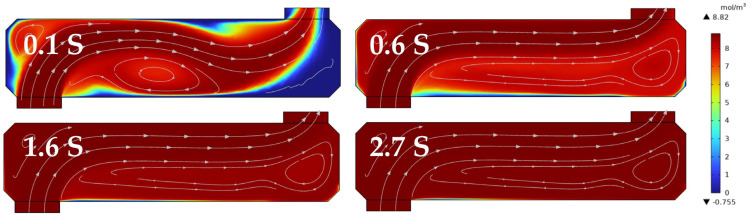
Internal fluid simulation and response time of a novel off−axis integral cavity structure.

The system’s response time is primarily governed by the volume of the gas chamber and the intake flow rate. To measure the response time, O_2_ or CO_2_ of varying concentrations was introduced into the integrating cavity. As illustrated, the Figure 10 and Figure 11 presents cross−sectional fluid simulations of different intake methods for two types of integrating cavity structures. The conventional intake structure of the integrating cavity, as shown in Figure 10, features an internal cavity length of 6.2 cm, a mirror size of 5.08 cm, and an air inlet positioned at the top of the cavity, with a gas flow rate set at 1 L/min. The simulation results indicate that, due to the influence of intake methods and structural characteristics, rapid air exchange within the cavity in less than 10 s leads to the formation of eddies and dead zones, which hinders fast response and precise measurement.

Hence, structural optimizations were made to the integrating cavity by eliminating the larger−sized high−reflection mirror (≥5.08 cm) commonly used in systems to reduce the size of the off−axis integrating cavity and thereby enhance the response time. In this study, a compact off−axis integrating cavity was designed, utilizing a 1−inch high−reflection mirror, with an overall cavity length of 7.6 cm, an inner diameter of 1.8 cm, and a distance of 6.2 cm between the two mirror surfaces. To mitigate the dead zone phenomenon during the air ventilation of the optical cavity, the mirror mounts were internally chamfered, and the intake method was altered to reduce the impact of eddies on the detection accuracy without affecting the reflection of the off−axis light beam. At a gas flow rate of 1 L/min, the theoretical calculation determined that it takes 2.873 s to fill the gas chamber. Using a 20% O_2_ concentration, the equilibrium time within the cavity was calculated at a flow rate of 1 L/min. The simulation results revealed that the airflow within the integrating cavity became gradually stable after 0.6 s, and the O_2_ concentration reached equilibrium after 2.7 s.

**Figure 12:**
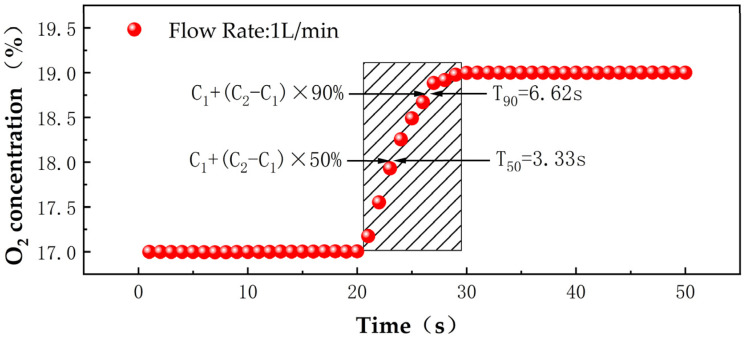
Measured O_2_ 17–19% instrument T_90_ response time.

The actual test results, as shown Figure 12, set the flow rate of the gas distributor at 1 L/min, initially introducing 10 min of low−concentration O_2_ (17%), with the average of the last 2 min taken as C_1_, followed by switching to high−concentration O_2_ (19%) for 10 min and the average of the last 2 min taken as C_2_. During the switching process, the response times for T_90_ were calculated as 6.62 s (T_2_−T_1_). The test results demonstrate the instrument’s excellent rapid response performance, enabling quick responses to changes in the concentration of the measured gas [24].

### 3.5. Filter Optimization

#### 3.5.1. Conventional Phase−Locked Amplification

Noise is a pivotal factor affecting the sensitivity and detection limit of gas detection. The traditional phase−locked amplifier is effective in extracting the target frequency signal while reducing the impact of noise. In gas detection, the amplitude of the secondary harmonic signal after gas absorption is directly proportional to the concentration of the gas being measured. Consequently, by mixing the input signal with a double−frequency reference signal and passing the output through a low−pass filter, the relative concentration of the gas can be obtained. Despite the minimization of circuit noise by the conventional phase−locked amplifier, variables such as external temperature, pressure fluctuations, and laser stability still introduce uncertainties that affect measurement accuracy.

#### 3.5.2. Windowed Fourier Transform (WFT)

Following the phase−locked amplifier, a low−pass filter is employed to demodulate the original signal. Traditional signal processing utilizes the Fourier transform, which lacks the ability to capture local characteristics and fails to analyze the relationship between frequency and time for non−stationary signals. In contrast, the Windowed Fourier Transform (WFT) divides the time−domain signal into equal short time intervals and performs Fourier transformations on each segment, thus obtaining frequency information across different time periods. However, Short−Time Fourier Transform does have limitations when selecting the window width.

#### 3.5.3. Wavelet Transform

Wavelet transform differs from Fourier transform by employing finite and decaying wavelet bases instead of infinite triangular function bases. The wavelet bases can be dilated and translated, providing both frequency information and temporal localization. Therefore, for the 2f signals detected using OA−ICOS technology, wavelet transform is particularly suitable for processing the signal amplitude in the time domain.

(1) $$(W\varphi f)(a,b)={\left|a\right|}^{-\frac{1}{2}}\int_{-\infty }^{+\infty }f(t)\varphi \left(\frac{t-b}{a}\right)dt$$

**Figure 13:**
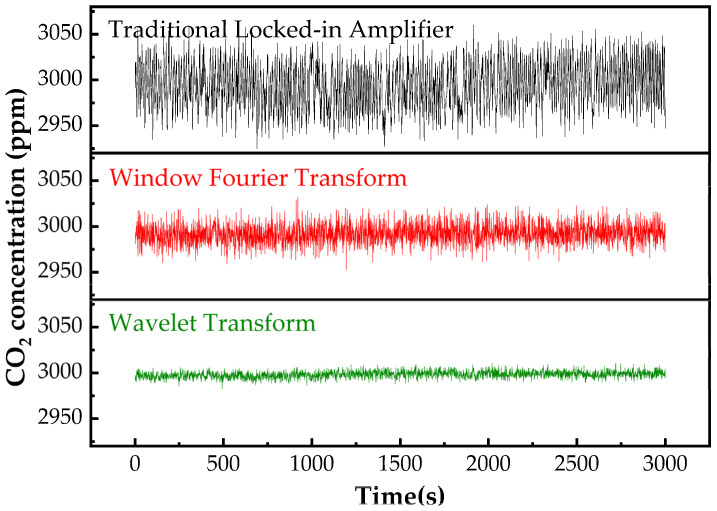
Analysis of 3000 ppm CO_2_ detection fluctuations using conventional phase−locked amplification, Windowed Fourier Transform (WFT), and wavelet transform with different noise reduction methods.

Here, Wφ represents the base function, f is the signal function, a is the scale factor, and b is the translation factor. By introducing a window length factor, the scale becomes variable, with a controlling the dilation of the base wavelet and b determining the time of signal analysis. The following Figure 13 illustrates the comparative results of the three filters. The signal fluctuation, after wavelet transform processing, intersected with the traditional phase−locked amplifier and windowed Fourier transform, showing reductions of 4.93 times and 2.83 times, respectively, with the standard deviation improved from 5.33 ppm to 1.21 ppm.

## 4. Field Application

Animals, after ingesting feed, undergo digestion and absorption to introduce nutrients into their bodies, which are then utilized in energy metabolism through biological oxidation−reduction reactions. This process essentially involves the animal inhaling O_2_, which enters the bloodstream through the lungs and is transported to various tissue cells, participating in oxidation−reduction reactions of nutrients and resulting in the production of CO_2_ and water. The CO_2_ is subsequently transported through the bloodstream to the lungs and exhaled. By measuring the amount of O_2_ consumed and CO_2_ exhaled by animals during this gas exchange process, we can indirectly calculate their heat production. The ratio of the amount of CO_2_ exhaled to the amount of O_2_ consumed, known as the respiratory quotient (RQ), can be used to assess the physiological state of the animal.

RQ = Emissions of CO_2_ (L)/Consumption of O_2_ (L) (2)

This study employed an open recirculation device with sensors to evaluate the concentrations of O_2_ and CO_2_ in the breath of poultry. Yellow chickens from Dehui City, Jilin Province, were reared in one group (Coop A) and another group (Coop B), respectively, and provided with ample feed. Continuous 24−h gas concentration monitoring was conducted using the natural feeding method. The gases were introduced in a series distribution, with the atmosphere first serving as the background gas for CO_2_ and O_2_ concentration calculations, followed by the extraction of the gas post−metabolism by the yellow chickens, which was filtered and dust−removed before entering the sensors. Since the intake flow rate of the sensors was constant, the mean value of the last minute with stable concentration values was taken as the measured concentration. Among the measured values there are four concentrations: C_coops_ is the concentration of CO_2_ in the chicken coops, C_atmosphere_ is the concentration of CO_2_ in the atmosphere, O_atmosphere_ is the concentration of O_2_ in the atmosphere, and O_coops_ is the concentration of O_2_ in the chicken coops. The respiratory entropy of chicken coops A and B at different times of the day were calculated separately using Equation (3).

RQ = (C_coops_ − C_atmosphere_)/(O_atmosphere_ − O_coops_) (3)

**Figure 14:**
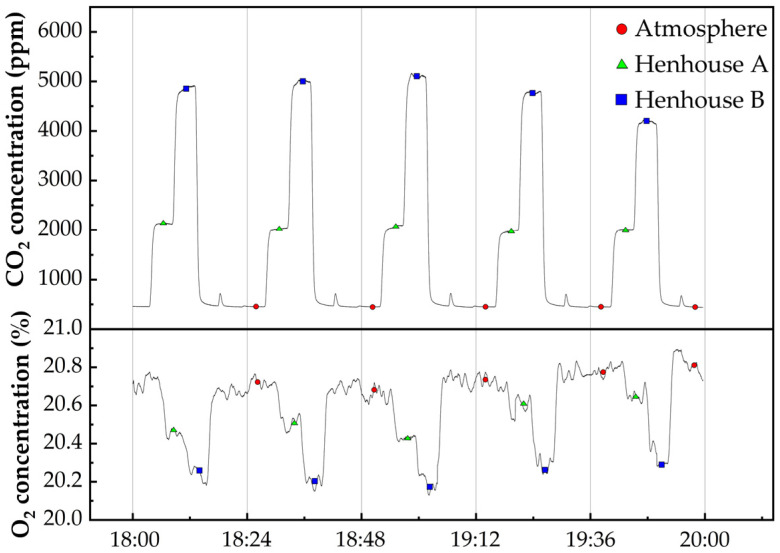
O_2_ and CO_2_ concentrations in chicken coop A and coop B from 6:00 to 8:00 p.m. Five sets of measured data.

**Figure 15:**
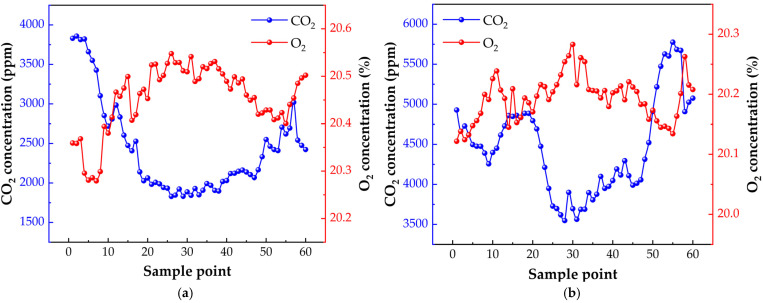
O_2_ and CO_2_ concentrations in Coops A (**a**) and B (**b**) over 24 h.

**Figure 16:**
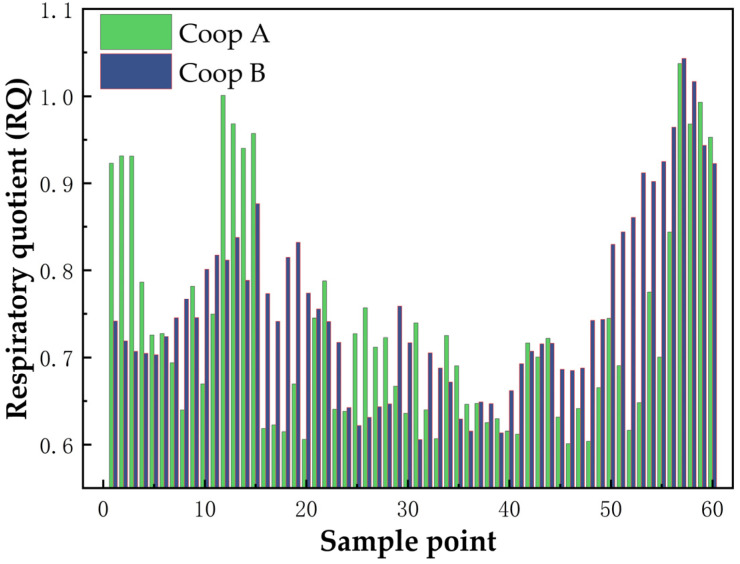
Respiratory quotient between Coop A and Coop B.

Figure 14 presents the measured data of O_2_ and CO_2_ concentrations within chicken coops from 6:00 to 8:00, across five experimental groups. The concentrations were assessed at 24−min intervals for the ambient air in Coop A and Coop B. One yellow chicken was placed in Coop A, while two were placed in Coop B. Continuous 24−h monitoring was conducted to track the gas concentration variations within these two coops. In the Figure 15, graphs a and b depict the 24−h patterns of gas concentration fluctuations for Coops A and B, respectively. The respiratory quotient (RQ) for both groups of chicks was calculated over the 24−h period using the respiratory quotient calculation formula. The results, as presented in the Figure 16, indicate that the RQ values align with the physiological activity patterns during feeding (RQ = 0.95–1.05) and fasting (RQ = 0.6–0.8) stages in chicks. This demonstrates the instrument’s suitability for the indirect calorimetry measurement of the avian respiratory quotient.

## 5. Conclusions

In this study, a self−developed dual gas sensor based on off−axis integrated cavity enhanced absorption spectroscopy was designed and measured the concentrations of O_2_ and CO_2_ for poultry RQ assessment. An innovative temperature and pressure regulation control module was developed to minimize the effects of environmental changes on gas measurement accuracy. To meet the demand for rapid response, a novel counterflow integral cavity structure was devised, effectively reducing the response time to 6.62 s (T_90_). The detection system achieved a minimum detection limit for O_2_ of 2.9 ppm at 622 s and carbon CO_2_ of 7.4 ppb at 350 s. The system underwent a 24−h field test in an open recirculating poultry house, where its reliability and stability were validated through theoretical computations based on poultry respiration entropy. This design offers a new detection method for rapid response and high−precision detection in the livestock industry.

## Figures and Tables

**Table 1:** Performance and cost comparison of different detection methods for O_2_ concentration.

Testing Method	Measurement Range	Accuracy	Response Time (T90)	Stability	Cost (RMB)
Electrochemical	0~100%	±3%FS	<60 s	one week	10,000
Gas Chromatography	0~30%	±1%FS	<120 s	one month	500,000
CRDS	5~25%	<2 ppm	<5 s	three months	1,950,000
OA−ICOS	0~25%	<100 ppm	<5 s	three months	100,000

## Data Availability

Data are contained within the article.

## References

[1] Barzegar S., Wu S.B., Noblet J., Choct M., Swick R.A. (2019). Energy efficiency and net energy prediction of feed in laying hens. Poult. Sci..

[2] Sharma N.K., Ban Z.B., Classen H.L., Yang H.M., Yan X.G., Choct M., Wu S.B. (2021). Net energy, energy utilization, and nitrogen and energy balance affected by dietary pea supplementation in broilers. Anim. Nutr..

[3] Li W., Wu T., Yan H., Gao M., Zhang K., Li Z. (2023). Monitoring of Atmospheric CH_4_ and CO_2_ by Off-Axis Integrating Cavity Output Spectra Based on RF White Noise. Acta Opt. Sin..

[4] Sun J.L., Jin C.J., Li L.S., Hong Y.R. (2001). A novel electrochemical oxygen sensor for determination of ultra-low oxygen contents in molten metal. J. Univ. Sci. Technol. Beijing.

[5] Lässer R., Grünhagen S., Kawamura Y. (2003). Use of micro gas chromatography in the fuel cycle of fusion reactors. Fusion Eng. Des..

[6] Fu B., Zhang C.H., Lyu W.H., Sun J.X., Shang C., Cheng Y., Xu L.J. (2022). Recent progress on laser absorption spectroscopy for determination of gaseous chemical species. Appl. Spectrosc. Rev..

[7] Shi M., Liu N. (2003). Development and prospects of ZrO_2_-based solid electrolyte oxygen sensors. J. Hefei Polytech. Univ. Nat. Ed..

[8] Chen H., Winderlich J., Gerbig C., Hoefer A., Rella C.W., Crosson E.R., Van Pelt A.D., Steinbach J., Kolle O., Beck V. (2010). High-accuracy continuous airborne measurements of greenhouse gases (CO_2_ and CH_4_) using the cavity ring-down spectroscopy (CRDS) technique. Atmos. Meas. Tech..

[9] Sargent B.J. (1989). A Transparent Oxygen Sensor Array.

[10] He Q.X., Li M.X., Lu H.Z., Li J.K. (2024). Environmental Oxygen Monitoring in Confined Spaces by a Mobile Sensor System Based on OA-ICOS and PSO-SVM Without Pressure Control. IEEE Sens. J..

[11] Yu R.Q., Xia H., Pang T., Wu B., Li Z., Sun P.S., Guo Q., Zhang Z.R., Cai Y.J. (2023). Simultaneous detection of CO_2_/CH_4_ based on off-axis integrated cavity output spectroscopy and time-division-multiplexing-based wavelength modulation spectroscopy. Opt. Commun..

[12] Lu J.C., Gao L., Wu Q., Liu W., Liang Y.J., Chen D.R., Shao J. (2024). Carbon Dioxide Measurement Based on Off- axis Integrated Cavity Output Spectroscopy Technology. Acta Photonica Sin..

[13] Wang J.N., Chuai Y.H., Li P.B., Lin G.Y. (2022). Near-Infrared Off-Axis Integrated Cavity Output Spectroscopic Dual Greenhouse Gas Sensor Based on FPGA for in situ Application. IEEE Access.

[14] Yuan Z.H., Huang Y.B., Lu X.J., Huang J., Liu Q., Qi G., Cao Z.S. (2021). Measurement of CO_2_ by Wavelength Modulated Reinjection Off-Axis Integrated Cavity Output Spectroscopy at 2 μm. Atmosphere.

[15] Gordon I.E., Rothman L.S., Hargreaves R.J., Hashemi R., Karlovets E.V., Skinner F., Conway E.K., Hill C., Kochanov R.V., Tan Y. (2022). The HITRAN2020 molecular spectroscopic database. J. Quant. Spectrosc. Radiat. Transf..

[16] Chang J.Q., He Q.X., Li M.X. (2023). Development of a stable oxygen sensor using a 761 nm DFB laser and multi-pass absorption spectroscopy for field measurements. Sensors.

[17] Zheng K.Y., Zheng C.T., Li J.H., Ma N.N., Liu Z.D., Li Y.Y., Zhang Y., Wang Y.D., Tittel F.K. (2020). Novel gas-phase sensing scheme using fiber-coupled off-axis integrated cavity output spectroscopy (FC-OA-ICOS) and cavity-reflected wavelength modulation spectroscopy (CR-WMS). Talanta.

[18] Han L., Xia H., Pang T., Zhang Z.R., Wu B., Liu S., Sun P.S., Cui X.J., Wang Y., Sigrist M.W. (2018). Frequency stabilization of quantum cascade laser for spectroscopic CO_2_ isotope analysis. Infrared Phys. Technol..

[19] Liu K., Wang L., Tan T., Wang G.S., Zhang W.J., Chen W.D., Gao X.M. (2015). Highly sensitive detection of methane by near-infrared laser absorption spectroscopy using a compact dense-pattern multipass cell. Sens. Actuator B-Chem..

[20] Sprenger M., Tetzlaff D., Soulsby C. (2017). No influence of CO_2_ on stable isotope analyses of soil waters with off-axis integrated cavity output spectroscopy (OA-ICOS). Rapid Commun. Mass Spectrom..

[21] Gonzalez-Valencia R., Magana-Rodriguez F., Gerardo-Nieto O., Sepulveda-Jauregui A., Martinez-Cruz K., Anthony K.W., Baer D., Thalasso F. (2014). In Situ Measurement of Dissolved Methane and Carbon Dioxide in Freshwater Ecosystems by Off-Axis Integrated Cavity Output Spectroscopy. Environ. Sci. Technol..

[22] Arévalo-Martínez D.L., Beyer M., Krumbholz M., Piller I., Kock A., Steinhoff T., Körtzinger A., Bange H.W. (2013). A new method for continuous measurements of oceanic and atmospheric N_2_O, CO and CO_2_: Performance of off-axis integrated cavity output spectroscopy (OA-ICOS) coupled to non-dispersive infrared detection (NDIR). Ocean Sci..

[23] Wang J.J., Tian X., Dong Y., Zhu G.D., Chen J.J., Tan T., Liu K., Chen W.D., Gao X.M. (2019). Enhancing off-axis integrated cavity output spectroscopy (OA-ICOS) with radio frequency white noise for gas sensing. Opt. Express.

[24] Hu J.Y., Lei H., Zhang H.Y., Xue X.X., Wang X.P., Wang C.H., Zhang Y. (2024). High reliable gas sensor based on crystal-facet regulated α-Fe_2_O_3_ nanocrystals for rapid detection of exhaled acetone. Rare Met..

